# Palliative Care Use Among People Living With HIV and Cancer: An Analysis of the National Cancer Database (2004-2018)

**DOI:** 10.1200/OP.22.00181

**Published:** 2022-07-22

**Authors:** Jessica Y. Islam, Leticia Nogueira, Gita Suneja, Anna Coghill, Tomi Akinyemiju

**Affiliations:** ^1^ Cancer Epidemiology Program, Center for Immunization and Infection in Cancer, H. Lee Moffitt Cancer Center and Research Institute, Tampa, FL; ^2^ Department of Population Health Sciences, Duke University, Durham, NC; ^3^ American Cancer Society, Atlanta, GA; ^4^ Deparment of Radiation Oncology, University of Utah, Salt Lake City, Utah

## Abstract

People living with HIV (PLWH) diagnosed with cancer are less likely to receive quality cancer treatment compared with HIV-negative patients. Timely provision of palliative care (PC) during cancer treatment can increase patient's survival and improve quality of life. Our objective was to compare the use of PC by HIV status among adults diagnosed with cancer in the United States.

**METHODS:**

More than 19 million individuals age 18-90 years diagnosed with the 11 most common cancers among PLWH were selected from the National Cancer Database (2004-2018). The National Cancer Database defined PC as any surgery, radiation, systemic therapy, or pain management treatment with noncurative intent. Multivariable logistic regression was used to examine associations between HIV status and PC receipt by cancer site and stage after adjustment for covariates.

**RESULTS:**

The study population included 52,306 HIV-positive (average age: 56.5 years) and 19,115,520 HIV-negative (average age: 63.7 years) cancer cases. PLWH diagnosed with stage I-III cancer were more likely to receive PC compared with their HIV-negative counterparts (adjusted odds ratio [aO]: 1.96; 95% CI, 1.80 to 2.14); however, they were also less likely to receive curative cancer treatment (aOR, 0.48; 95% CI, 0.40 to 0.59). PLWH diagnosed with stage IV cancer were less likely to receive PC (aOR, 0.70; 95% CI, 0.66 to 0.74) compared with HIV-negative patients. When evaluated by cancer site, PLWH diagnosed with stage IV lung (aOR, 0.80; 95% CI, 0.73 to 0.87) and colorectal (aOR, 0.72, 95% CI, 0.54 to 0.95) cancers were less likely to receive PC than HIV-negative patients.

**CONCLUSION:**

PLWH diagnosed with stage IV cancer, particularly lung and colorectal cancers, were less likely to receive PC compared with cancer patients without HIV. PLWH with nonmetastatic disease were more likely to receive PC but less likely to receive curative treatment, reinforcing that clinical strategies are needed to improve the quality of care among PLWH.

## INTRODUCTION

People living with HIV (PLWH) experience elevated mortality for several cancers compared with HIV-negative persons^1^ because of multiple factors including HIV-related immunosuppression, which impairs control of oncogenic viral infections,^2^ and social determinants of health affecting cancer care delivery.^3^ Poorer survival is not limited to cancers with a viral etiology, and previous studies have shown that worse outcomes among PLWH persist after adjustment for differences in patient demographics and cancer stage.^4^ Among other factors, differences in cancer treatment delivery may contribute to worse survival among PLWH.^5^

In the United States, PLWH with cancer are less likely to receive any cancer treatment compared with their HIV-negative counterparts.^4,6,7^ For example, a study conducted using 2003-2011 data from the National Cancer Database (NCDB) found that patients with cancer and HIV were less likely to receive any modality of curative cancer treatment compared with patients without HIV for several common cancer types (7). Disparities in high-quality cancer care delivery among PLWH are likely due to multifactorial causes from several perspectives, including patients and providers.^5^ Physicians may choose to withhold treatment from PLWH with cancer because of concerns regarding side effects or medication interactions and general perception of frailty among PLWH. A survey of US oncologists demonstrated that physicians believed cancer patients with HIV were more likely to have treatment toxicity or decreased efficacy of cancer therapy because of interactions with their HIV treatment and were therefore less likely to offer standard cancer treatment to PLWH.^8^ From the patient perspective, qualitative interviews with cancer patients with HIV have revealed that common barriers to access to cancer care include stigma surrounding HIV, challenges with care accessibility, such as parking or transportation to their cancer treatment facility, and issues coping with mental health.^3^

To address these documented barriers, recent National Comprehensive Cancer Network (NCCN) guidelines have been developed to recommend that PLWH should be offered the same therapy as cancer patients without HIV.^9^ In addition to guidelines regarding curative therapy, the HIV and cancer NCCN guidelines included recommendations toward the use of palliative care (PC) among PLWH to ensure equitable delivery of supportive care during cancer treatment regardless of HIV status. For the general cancer patient population, the NCCN clinical practice in oncology guidelines recommends timely and early intervention with PC consults.^10^ Timely provision of PC after cancer diagnosis through an integrated care model can improve quality of life, including alleviating pain associated with cancer treatment and adverse mental health outcomes, such as symptoms of depression. Early intervention with PC increases survival among patients with advanced cancer.^11-15^ Benefits also include higher satisfaction with cancer care and fewer patients receiving unnecessary invasive measures at the end of life. The benefits of PC are particularly salient in the context of HIV, given that PLWH are more likely to experience a high burden of poor mental health outcomes, including depression, because of several factors including internalized HIV-related stigma,^16,17^ experiences of discrimination in the health care setting,^18^ the reality of living with a chronic condition, and social inequities that disproportionately burden PLWH in the United States.^19^ Therefore, provision of PC is an important component of quality cancer care among PLWH.^20^ However, research describing the use of PC among PLWH with cancer is unavailable.

In our current study, we used data from the US NCDB to assess differences in PC use among individuals diagnosed with the 11 cancer sites that commonly occur among PLWH by HIV status. To our knowledge, this is the first and largest study to date examining differences in the receipt of PC by HIV status among patients with cancer in the United States.

## METHODS

### Data Source

The NCDB is a hospital-based cancer registry jointly sponsored by the American College of Surgeons and the American Cancer Society, which captures approximately 72% of all cancer cases in the United States from more than 1,500 facilities accredited by the American College of Surgeons' Commission on Cancer.^21^ Data reported to the NCDB are abstracted by Certified Tumor Registrars^22^ who use standardized methods to collect sociodemographic and clinical data, including tumor type, stage, grade, and receipt of cancer treatment. To ensure high-quality and accurate data, the data are standardized according to national standards and Commission on Cancer–accredited sites undergo an external review of hospital charts and registry abstracts of at least 10% of records every 3 years.^23^ The study was approved by the Duke University Institutional Review Board under a general study protocol (IRB#: Pro00102834) for analyses using NCDB data. As the NCDB is a deidentified data set, this study was granted exemption.

### Study Cohort

Individuals diagnosed between 2004 and 2018 with the 11 most common cancers^2^ among PLWH were selected, including Kaposi Sarcoma, cancers of the head and neck (oral cavity, pharynx, and larynx), upper GI tract (pancreas, stomach, and esophagus), colorectum, anus, lung, female breast, cervix, and prostate; Hodgkin lymphoma; and diffuse large B-cell lymphoma (DLBCL). Cancer sites were identified using the SEER cancer statistics review using International Classification of Diseases for Oncology, 3rd Edition (ICD-O-3) site and histology codes.^24^ Cancer stage was categorized according to the American Joint Committee on Cancer staging.^25^ HIV status was determined from reported comorbidities using the ICD-9-CM diagnosis codes 04200-044.90, 07593, and V0800 and ICD-10-CM codes B20-B22, B24, and Z21.

### Measures

The primary outcome was PC use as defined by the NCDB.^26-30^ The NCDB includes information on any PC from patients' medical records provided during their treatment at the reporting facility. The NCDB codes treatment as palliative only if the patient's medical records explicitly mentioned that the goal of treatment is palliation and not cure. Specifically, procedures were categorized as PC if treatment was provided to prolong a patient's life by controlling symptoms, to alleviate pain, or to make the patient more comfortable.^31^” Types of PC included pain management therapy, surgery, radiation therapy, or systemic chemotherapy administered to alleviate symptoms. Patients using PC in the NCDB may concurrently be undergoing curative treatment. The NCDB does not document hospice services or referral, and thus, hospice was not included in the definition of PC.

Receipt of curative cancer treatment was defined as surgery, radiotherapy, systemic therapy, or any combination of these therapies for all cancer sites excluding DLBCL. First course curative treatment for DLBCL was defined as chemotherapy, radiotherapy, or a combination of both. Patient characteristics included age at diagnosis, sex, race/ethnicity, insurance status, area-level income level, type of cancer treatment facility, census region, and year of cancer diagnosis. Race/ethnicity was defined as non-Hispanic (NH) White, NH Black, Hispanic, and Others. Zip code–level median income was categorized into quartiles on the basis of data provided by the American Community Survey.^32^ Insurance status was determined according to coding for primary payer at diagnosis and was categorized as Private, Medicaid, Medicare, Uninsured, or Others. Facility type was categorized as Community Cancer Program, Comprehensive Community Cancer Program, Teaching/Academic Research Program, National Cancer Institute Program/Network, and Others. HIV/AIDS is one of 15 noncancer comorbid conditions in the Charlson-Deyo comorbidity score, and therefore, we recalculated the comorbidity score excluding HIV/AIDS to derive a modified Charlson-Deyo comorbidity score independent of HIV infection to reflect non–HIV-related disease burden.^7^ Patient comorbidities were identified according to the modified Charlson-Deyo comorbidity index and categorized into 0, 1, or ≥ 2.^33^

### Statistical Analysis

We conducted descriptive analyses using chi-square univariate comparisons of patients' characteristics by HIV status. We used multivariable logistic regression to evaluate the associations between HIV status and receipt of PC overall and by cancer site. The models were adjusted for variables deemed relevant (possible confounders) a priori on the basis of expert knowledge and included the following: age at diagnosis, race/ethnicity, sex, insurance, geographic region, comorbidity index, and cancer diagnosis year.^34^ We used the same adjustment set when we stratified the models by cancer site and stage at diagnosis. Finally, we compared the use of curative treatment among those diagnosed with stage I-III cancers by HIV status using multivariable logistic regression overall and stratified by use of PC. On the basis of the exploratory and descriptive nature of this analysis, we did not include an adjustment for multiple comparisons for data presentation.^35,36^ All analyses were performed using SAS 9.4. Statistical significance was set at 2-sided α = .05.

## RESULTS

More than 19 million individuals diagnosed with cancer between 2004 and 2018 were selected from the NCDB, including 52,036 PLWH and 19,115,520 individuals without a HIV (Table 1). PLWH with cancer were younger, more likely to be male, insured by Medicaid, resided in lower-income areas, and less likely to be White compared with their HIV-negative counterparts. Lung cancer was the most commonly observed cancer among both PLWH and HIV-negative adults. Additional cancers frequently diagnosed among PLWH included DLBCL (16% of cancers in PLWH); Kaposi Sarcoma (11%); and colorectal (11%), anal, and prostate (8%) cancers. PLWH were more likely to be diagnosed at stage IV compared with HIV-negative adults (32% *v* 19%, *P* < .001).

**TABLE 1.**
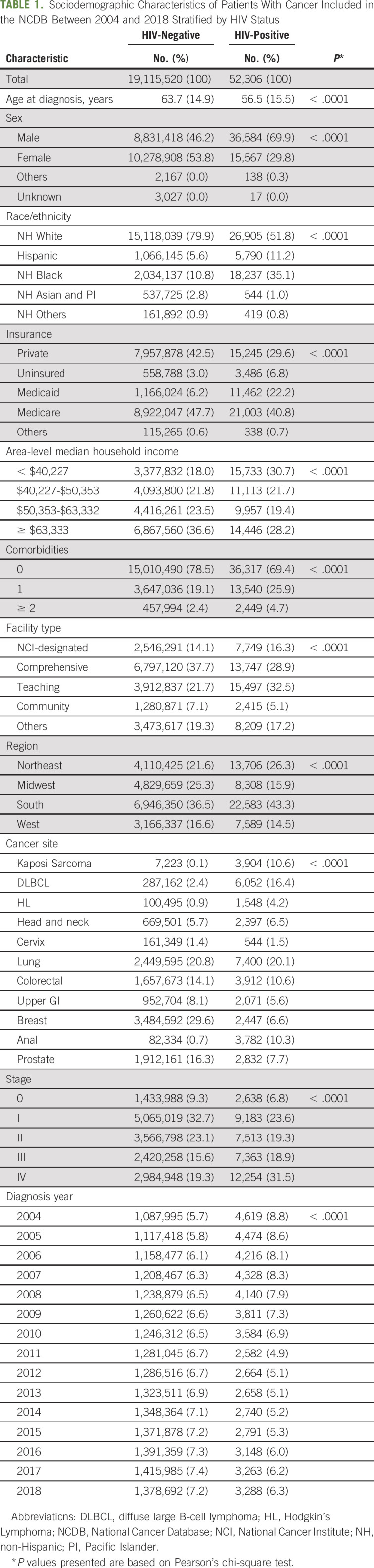
Sociodemographic Characteristics of Patients With Cancer Included in the NCDB Between 2004 and 2018 Stratified by HIV Status

Overall, 5% of PLWH with cancer received PC (Table 2). PLWH were 25% more likely to receive PC compared with their HIV-negative counterparts (adjusted odds ratio [aOR]: 1.25; 95% CI, 1.20 to 1.30) after adjustment for age, sex, race/ethnicity, insurance type, geographic region, modified comorbidity score, and year of cancer diagnosis. When we stratified by stage (I-III *v* IV), we observed that PLWH diagnosed with stage I-III cancer were 96% more likely to receive PC compared with HIV-negative patients (aOR, 1.96, 95% CI, 1.80 to 2.13). By contrast, PLWH diagnosed with stage IV cancer were 30% less likely to receive PC than cancer patients without HIV (aOR, 0.70; 95% CI, 0.66 to 0.74).

**TABLE 2.**
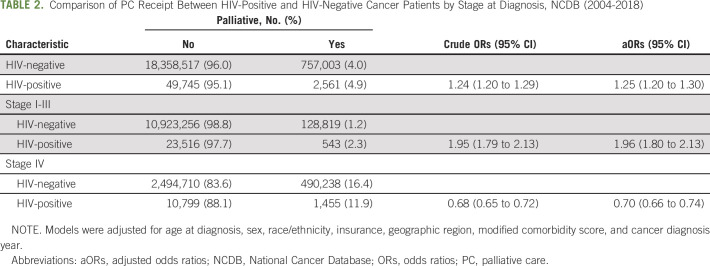
Comparison of PC Receipt Between HIV-Positive and HIV-Negative Cancer Patients by Stage at Diagnosis, NCDB (2004-2018)

Patients with DLBCL and HIV were more likely to receive PC compared with their HIV-negative counterparts for all stages of cancer diagnosis (Table 3). Patients with stage I-III head and neck (aOR, 2.55; 95% CI, 1.65 to 3.94), breast (aOR, 2.16; 95% CI, 1.22 to 3.82), anal (aOR, 1.51; 95% CI, 1.12 to 2.05), and colorectal (aOR, 1.76; 95% CI, 1.25 to 2.49) cancers were more likely to receive PC compared with HIV-negative patients diagnosed with the same cancer site and stage, even after adjustment for covariates. By contrast, for metastatic disease (stage IV), patients diagnosed with colorectal cancer were less likely to receive PC (aOR, 0.72; 95% CI, 0.54 to 0.94) compared with their HIV-negative counterparts. Similarly, patients diagnosed with stage IV lung cancer had 20% lower odds of receiving PC (aOR, 0.80; 95% CI, 0.73 to 0.87) compared with those without HIV.

**TABLE 3.**
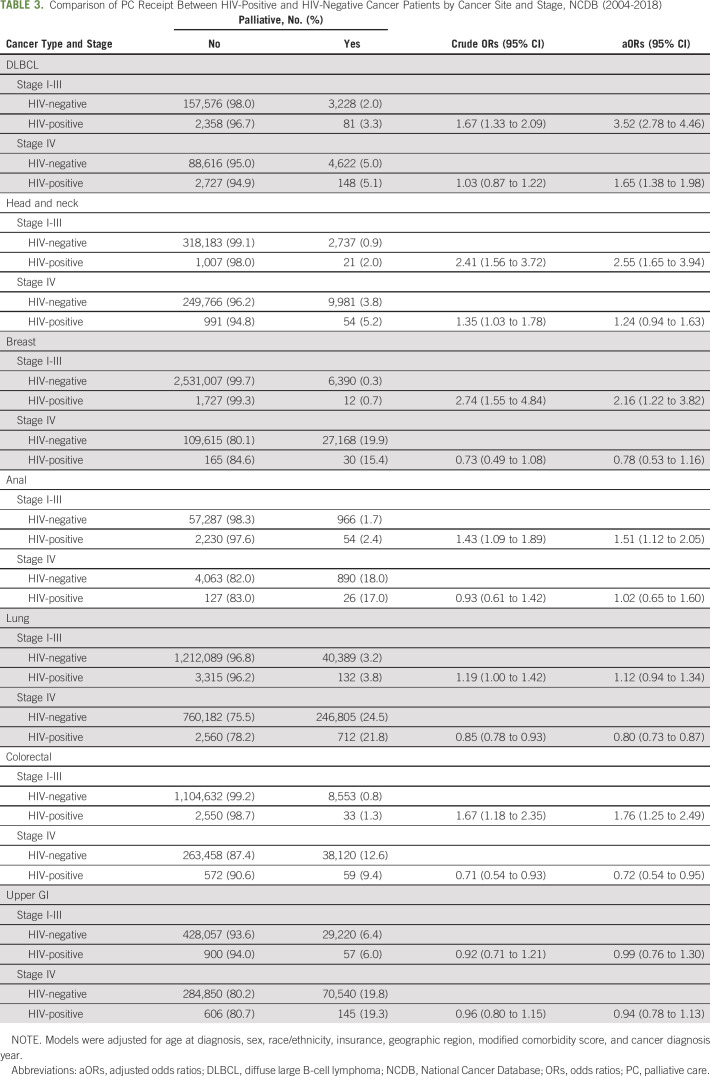
Comparison of PC Receipt Between HIV-Positive and HIV-Negative Cancer Patients by Cancer Site and Stage, NCDB (2004-2018)

Among individuals diagnosed with stage I-III cancers, PLWH were less likely to receive any curative treatment (aOR, 0.51; 95% CI, 0.49 to 0.53) compared with their HIV-negative counterparts, regardless of PC receipt (Table 4). PLWH who received PC were 48% less likely to receive curative treatment and PLWH who did not receive PC were 52% less likely to receive curative cancer care than cancer patients without HIV.

**TABLE 4.**
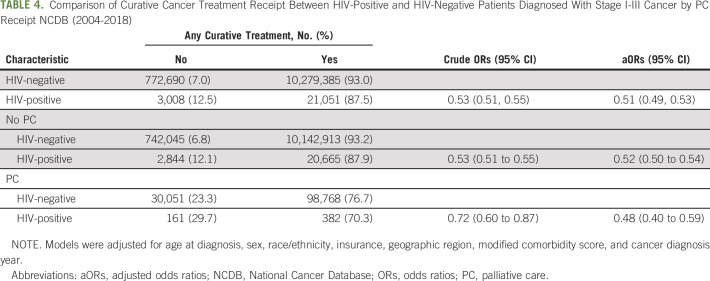
Comparison of Curative Cancer Treatment Receipt Between HIV-Positive and HIV-Negative Patients Diagnosed With Stage I-III Cancer by PC Receipt NCDB (2004-2018)

## DISCUSSION

Overall, use of PC was low among PLWH, with only one in 20 cancer patients living with HIV receiving palliative care in the United States. Despite the documented benefits of PC in survival and quality of life among cancer patients with metastatic disease,^37^ we observed disparities in PC use particularly among HIV-positive cancer patients diagnosed with stage IV malignancies specifically patients with lung and colorectal cancer. Although PLWH diagnosed with stage IV cancer are less likely to receive PC, compared with those without HIV, our study suggests that PLWH diagnosed with stage I-III cancer are more likely to receive PC in lieu of curative therapy. This is concerning given the NCCN guidelines for administration of early PC concurrent with curative therapy for people with stage I-III cancers and PC for all people with stage IV cancers. These results reinforce previous research demonstrating disparities in receipt of curative treatment among PLWH,^4^ underscoring an opportunity to improve cancer care across the continuum from diagnosis to end of life.

In our study of more than 19 million adults diagnosed with the most common cancer sites among PLWH in the United States, PLWH diagnosed with stage IV cancer were less likely to receive PC compared with cancer patients without HIV. These documented disparities among HIV-positive cancer patients with metastatic disease is particularly important given the potential benefits of PC in the HIV setting. PLWH with cancer experience a disproportionate burden of barriers to care because of documented experiences related to discrimination and stigma because of their HIV status.^3,17^ These societal barriers to care can lead to poorer cancer outcomes, such as higher rates of mortality, and adverse mental health symptoms such as stress, depression, and anxiety.^38^ Palliative care can directly address these adverse outcomes as the overall goal of PC is to anticipate, prevent, and reduce physical and psychosocial suffering, regardless of the stage of disease or curative treatment plans.^39^ In addition, PC is used to manage adverse physical symptoms that patients experience during cancer treatment such as dizziness, nausea, vomiting, loss of appetite, and overall pain. As outlined by the NCCN guidelines, PC consults should begin at cancer diagnosis and continue over the course of the cancer care continuum.^39^ The value of early integration of PC into cancer care has been demonstrated through improvements in symptom management, reduction in psychosocial distress, and enhancements in treatment decision making for patients.^40-45^

Our study demonstrates that PLWH diagnosed with stage IV cancers were less likely to receive PC compared with patients without HIV. However, patients living with stage IV cancers can experience the largest benefit from PC by mitigating several adverse symptoms including nausea, pain, vomiting, and poor mental health,^46^ as previously demonstrated among patients with non–small-cell lung cancer.^37,44^ Surprisingly, PLWH diagnosed with stage IV lung cancer were less likely to receive PC compared with lung cancer patients without HIV. Only one in five PLWH diagnosed with stage IV lung cancer received PC. In our study population, lung cancer was the most common cancer diagnosed among PLWH and lung cancer is the most frequent cause of cancer-related death among PLWH.^2,47,48^ The benefits of early intervention with PC among patients with cancer were first demonstrated among patients with metastatic non–small-cell lung cancer through a randomized controlled trial,^37^ which reported increased survival among patients who received PC. Given the absence of guidelines during the study period, numerous provider-level factors, including stigma associated with treating patients with HIV,^3^ likely contributed to lowering PC use among patients with stage IV lung cancer. Provider perceptions regarding the role of HIV in treatment adherence and efficacy and comfort level with discussing adverse cancer treatment effects and prognosis have been found to negatively affect likelihood of offering standard curative treatment to PLWH and cancer.^8^ Inequities in cancer care delivery among HIV-positive cancer patients have been previously documented leveraging various patient population registries and health care system contexts, including the NCDB,^49,50^ SEER-Medicare,^51,52^ and the HIV and Cancer Match Study.^4,6^ These studies have consistently documented that PLWH with cancer are less likely to receive any cancer treatment (defined as curative chemotherapy, radiation, or surgery) compared with their HIV-negative counterparts, even after adjustment for stage of cancer diagnosis and type of cancer. In the present study, we were interested in the interplay of curative and PC treatment receipt, and as such, we further evaluated whether this patient population living with HIV and cancer received standard curative care as recommended by NCCN Guidelines Panel for Cancer in People Living with HIV.^53^ We found that PLWH with stage I-III cancer at diagnosis were less likely to receive curative treatment compared with cancer patients without HIV, including those who received PC as part of their care. In fact, almost one third of PLWH with stage I-III cancer who received PC were not offered any form of curative cancer treatment. A previous study conducted using the NCDB demonstrated that PLWH were more likely to be left untreated compared with those without HIV across cancer sites, even after adjustment for insurance status and medical comorbidities.^7^ Our observation of a persistent disparity in curative treatment receipt among patients who are more likely to receive supportive care suggests that PLWH diagnosed with cancer may be solely receiving supportive care to alleviate symptoms attributable to their comorbid conditions rather than the side effects of cancer treatment. However, stage-appropriate cancer treatment can improve survival and cancer outcomes. Withholding cancer treatment, both curative and palliative, can lead to loss of quantity and quality of life, leading to downstream impacts on the social and economic livelihood of HIV-positive patients and their families. HIV status should not guide cancer treatment guidelines, and PLWH should have equal access to guideline-adherent, high-quality, cancer care.

Palliative care use is influenced by several factors including patient-level and provider-level characteristics. We are limited to the data available in the NCDB and are not able to evaluate unmeasured factors that influence physician’s or patient’s choices regarding PC. For example, patients may opt against PC because of personal choice or beliefs regarding end-of-life care.^54^ It is also important to contextualize the demographic distribution of PLWH in the United States. Similar to what we observed in our patient population, PLWH in the United States include majority NH Black, lower income, with a high proportion of Medicaid-insured patients.^55^ Health care system–level factors because of structural racism^56^ likely play a significant role in the disparities that we observed in palliative and curative cancer treatment delivery among PLWH. Future research focused on delineating the potential reasons for disparities that we observed in this study from a heath care system–level perspective is needed to inform the development of interventions to alleviate disparities in equitable cancer treatment delivery in the context of HIV and cancer. It is also important to acknowledge that the NCDB data on PC services are of uncertain accuracy and may be underascertained as hospice use is not documented. Palliative intent must be inferred from clinical records, and there is therefore an opportunity for misclassification of PC use and type of PC treatment. Although the NCDB captures 70% of patients with cancer in the United States, data are not population-based. Thus, certain patients who did not receive care at Commission on Cancer–accredited US hospitals have been under-represented. Nonetheless, to our knowledge, the present study is the largest analysis to date with a patient population that reflects the underlying demographics of the US HIV population to evaluate PC use in the context of cancer care. Future work evaluating the potential benefits of PC among people living with HIV and cancer should focus on important survival outcomes including overall survival and progression-free survival and time from cancer diagnosis to death, particularly by stage at cancer diagnosis.

In conclusion, our study highlights important opportunities for improving equitable cancer care delivery among patients living with HIV and cancer. Our present study, to our knowledge, is the first characterization of PC use among people living with HIV and cancer. Despite the documented benefits of PC, including improved survival and patient-reported quality of life, we observed concerning patterns for PLWH regardless of cancer stage. Importantly, although PLWH diagnosed with stage I-III were more likely to receive PC, they were also less likely to receive curative treatment, suggesting that supportive care may be offered in lieu of curative-intent therapy to PLWH with cancer. In the general oncology population (HIV-negative), integration of patient-reported outcome measurements into regular clinical care has improved symptom management.^57^ Patient-reported outcomes are defined by the National Cancer Institute as information directly from the patient to describe how they feel and function, such as pain or other symptoms, their satisfaction with care, and how a disease or treatment affects their physical, mental, emotional, spiritual, and health-related quality of life.^58^ Given that clinicians miss about half of their patients' symptoms during treatment,^59,60^ systematic monitoring of patients' symptoms using patient-reported outcomes may contribute to closing the gap in cancer care delivery in the context of PLWH's supportive or PC during cancer treatment.^57^ Future work focused on integrating the measurement of patient-reported outcomes or adverse symptoms that PC traditionally addresses during cancer care should be prioritized to inform the development of potential strategies to address inequities in palliation. Equitable access to PC is an important component of high-quality cancer care in the United States, and efforts to improve the delivery of PC to PLWH using insights from our analysis should be prioritized.

## Data Availability

The data that support the findings of this study are available on request from the corresponding author. The data are not publicly available due to privacy or ethical restrictions.
